# Supplementary material for: Use of a Polymer Inclusion Membrane and a Chelating Resin for the Flow-Based Sequential Determination of Copper(II) and Zinc(II) in Natural Waters and Soil Leachates
Source: Molecules. 2020 Oct 31;25(21):5062. doi: 10.3390/molecules25215062 (PMC7662993; doi:10.3390/molecules25215062)
Supplement: Supplementary file 1 [file molecules-25-05062-s001.pdf]

Electronic Supplementary Information

Use of a Polymer Inclusion Membrane and a Chelating Resin for the Flow-Based Sequential Determination of Copper(II) and Zinc(II) in Natural Waters and Soil Leachates

Tânia C. F. Ribas [1], Charles F. Croft [2], M. Inês G. S. Almeida [2], Raquel B. R. Mesquita [1], Spas D. Kolev [2] and António O. S. S. Rangel [1,*]

[1] Universidade Católica Portuguesa, CBQF - Centro de Biotecnologia e Química Fina – Laboratório Associado, Escola Superior de Biotecnologia, Rua Diogo Botelho 1327, 4169-005 Porto, Portugal
[2] School of Chemistry, University of Melbourne, VIC, 3010, Australia
* Correspondence: arangel@porto.ucp.pt

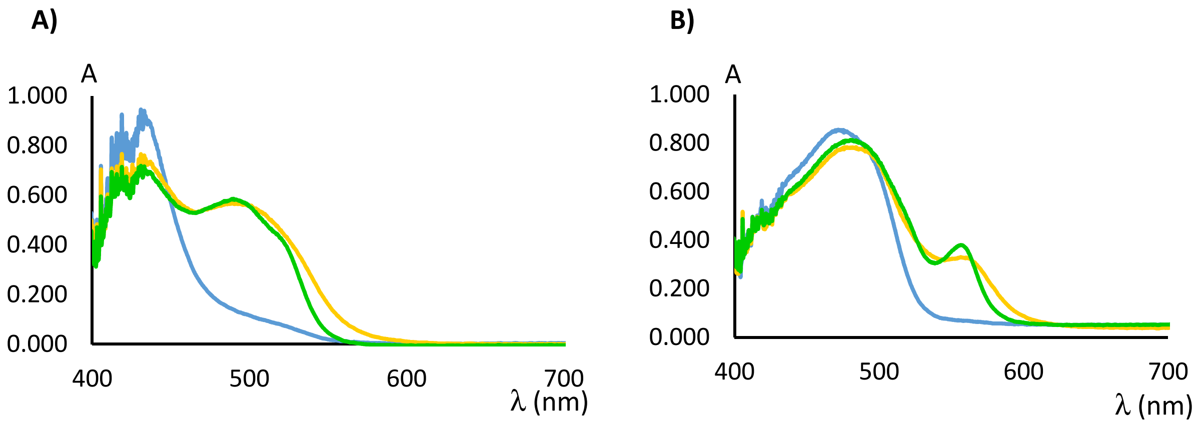

Figure S1. Spectra of the colour metal complexes with PAR (A) and PAN (B); spectra of the blank (reagent in milliQ water) (blue lines), Cu(II)-PAR/PAN complex (yellow lines) and Zn(II)-PAR/PAN complex (green lines); PAR/PAN concentration of 0.1 $mmolL^{-1}$; metal ion concentration of 0.5 μgL^{-1}; and carbonate buffer (0.6 $mmolL^{-1}$) solution at pH = 10.

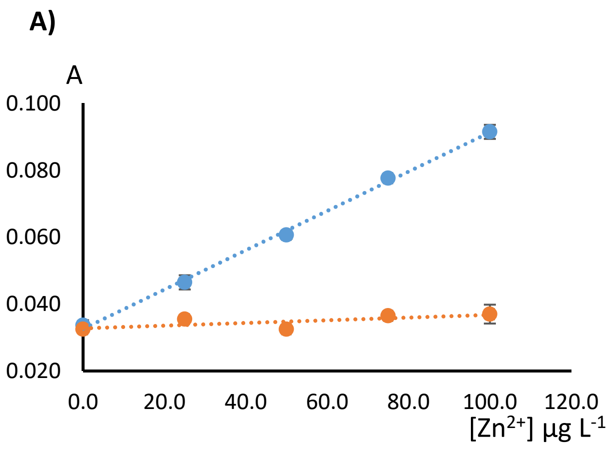

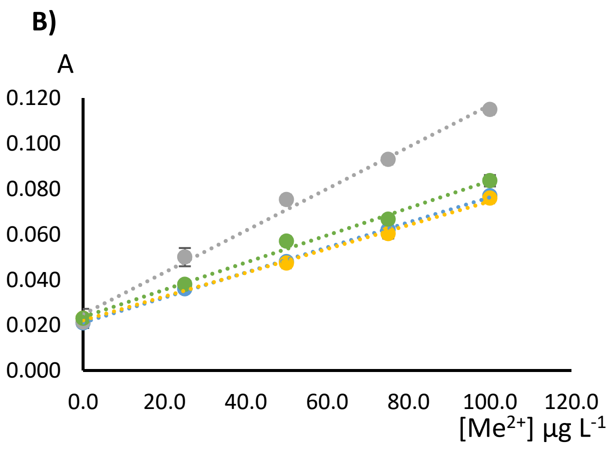

Figure S2. Study of the effect of the PIM column (A) and the Chelex column (B) on the calibration curves of zinc(II) and copper(II): (A) direct zinc(II) calibration curve without (blue) and with (orange) using a PIM; and (B) calibration curve with mixed standards of copper(II) and zinc(II) aspirated through the Chelex column (green) and without going through the Chelex column (grey) and calibration curve with zinc(II) standards with (yellow) and without (blue) using a Chelex column.

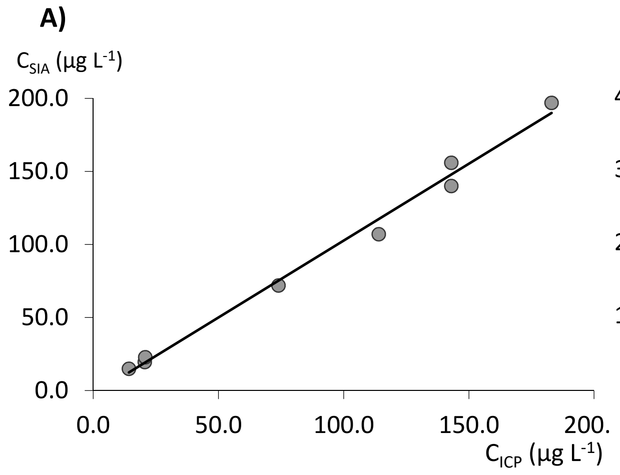

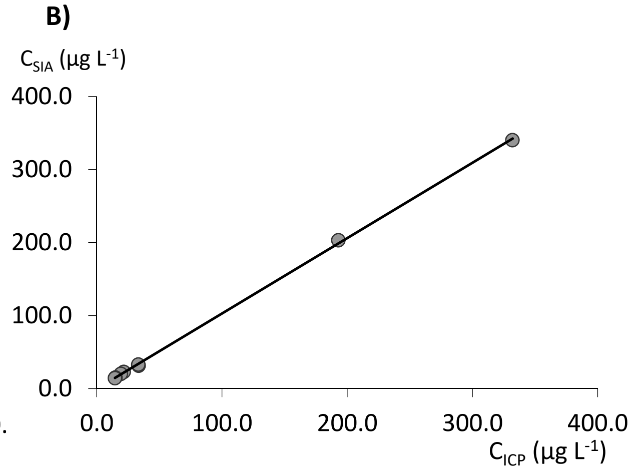

Figure S3. Comparison of the results obtained with the newly developed SIA system and those obtained with a reference method (ICP-OES): (A) copper(II) determination; and (B) zinc(II) determination. The lines represent the linear relationship between the two methods.
